# On Renal Inflammation

**Published:** 1823-08

**Authors:** 


### Art. III.—*On Renal Inflammation.* By Dr. Kinglake.

Renal inflammation is an affection, under any circumstances, of a very serious nature. Organs, like those of the kidneys, of vital importance to the existence and well-being of the animal economy, cannot have their function either suspended or disturbed, without materially disordering health, and even endangering life. The kidneys possess in the animal frame a situation of great local security, externally fenced for the most part by a bony fabric, and internally sheathed in a capsule, and often a fatty covering, affording to them at once an uniform temperature and an immunity from hurtful pressure. The ailments occurring in these organs are more likely to proceed from disorders imperceptibly arising in their excitability, structure, or function, than from mechanical or accidental causes.

The excitability of the kidneys, in common with that of every other organ connected with some special function in the animal economy, is generated by, and emanates from, the peculiar structure by which they are constituted. If the structure be distempered, its excitable product must share in such distemper: if it be disorganised, the effect on its excitable powers must correspond with such altered structure. Both the peculiarity of texture and excitability of the kidneys are subservient to the renal function, which is that of abstracting from the circulating fluids superfluous water, and the various excrementitious substances contained in it. This unremitting function, like that of the heart and lungs, cannot be impeded or deranged without inducing much disorder in the lungs themselves, as well as throughout the whole frame.

The providence of nature is admirably manifested in the multiplied and extended bulk given to the vital organs, whose functions could not be disturbed without endangering life. The large and diversified fabric of the kidneys, lungs, liver, and brain, are striking instances of this ample provision, by which, though a portion of their respective structures might be unfitted by disease for natural use, yet the general function may still for a time be tolerably well performed. Sudden deaths, and eventual destruction, from accidental diseases, are happily obviated by the voluminous resources of nature on these occasions. Hence it often occurs that the kidneys, like the lungs, and more especially the liver, will suffer, not only in their excitable property, but also in their very structure, causing large portions of it to sink into irreparable disease, without either destroying life or giving early notice of the existence of such extent of malady.

The early stages of all visceral inflammations are marked by symptoms denoting the degree of the existing affection; but this morbid violence is not lasting, nor is it indicated by sensations that would infer its presence. The diseased or inflamed portions may go into either suppurative decomposition or tuberculated induration, that may alike destroy natural sensibility, inducing a deceitful calm in the process of disease. The kidneys are perhaps more particularly liable to this insidious course of morbid spoliation. Instances have often occurred of the renal organs having been mutilated almost to annihilation by the devastating ravages of inflammation, and that too, for the most part, secretly and unexpectedly.

Two cases of the description referred to have fallen under my observation, worthy of being recorded, both as interesting objects of disease, and as suggesting the sort of treatment that would probably have obviated such fatal results.

A middle-aged woman, distinguished by no peculiarity of temperament, had experienced for a long time (nearly two years, if my recollection be correct,) considerable inconvenience about the region of the kidneys. The pain was described to have been at first very severe, afterwards as being comparatively moderate, but never altogether ceasing. The urinary secretion continued to be almost natural; nor was the general health materially affected, after the pain had become much abated. In the course of the complaint, the patient was admitted into the Exeter Hospital; where, after remaining long enough to ascertain that no benefit could be obtained, she was dismissed as incurable. She was afterwards received into the Taunton Hospital. At that time the abdomen was unnaturally protuberent and hard, but did not, on examination, appear to contain any fluid. A sense of morbid fullness and distention was more particularly referred to the region of the kidneys, stretching along the left side of the abdomen. The whole enlargement was either unyieldingly hard or remittent on pressure, but not acutely painful. An uneasy sensation was constantly experienced, in a greater or less degree. The general volume of the abdominal enlargement had the external character of ascital dropsy; but no fluctuation could, by the most attentive search, be discovered. Various speculations were held respecting the nature of this protuberance; amongst others, were those of its being either an ovarial, splenitic, pancreatic, mesenteric, or hepatic tumefaction. The morbid circumstances of this case continued to be nearly stationary until within a few days of the patient's death, which was preceded by loss of appetite, prostration of strength, and an accelerated action of the heart and arteries.

The day after the patient's decease the body was opened. The abdominal viscera, as they presented *in situ*, were apparently in a natural state; at least, not perceptibly enlarged. On proceeding to inspect the kidneys, much surprise was occasioned by the appearance of a membranous bag, filled with a fluid, occupying the situation of the left kidney, and greatly exceeding in dimensions the natural bulk of that organ. On dividing this bag, an aqueous fluid escaped, amounting probably to two or three pints, from its cavity. It appeared at first to be a membranous cyst of morbid formation; but, on laying it entirely open, and examining accurately its source and connexions, it proved to be the natural capsule of the kidney, and that its evidently contained kidney had been broken down and annihilated by disease; not a vestige of its substance remaining in the capsule that should have naturally enclosed it. Suppurative inflammation had most probably decomposed its whole body, and left its containing membrane or capsule as the reservoir of its wreck, and also of such exhalations as might have been supplied from its interior surface, and have accumulated in its cavity.

This case affords an instructive instance of the origin, progress, and termination, of a disease of the renal structure. It shows that inflammatory ailment, unsubdued, might train on to an untoward and irreparable course of disease. It further proves that the excitability of the kidneys will admit of being disturbed by morbid action, even to demolishing the very structure by which it is generated; and that the urinary secretion, being vested in duplicate organs, may be suspended and destroyed in one, whilst the necessary function will be tolerably well performed by the other. If absorption had wholly removed the decomposed and residual wreck of the substance of the kidney, it is probable that its containing capsule would have remained dormant, and would not, by morbid extension, have occasioned the death of the patient. The dissolved kidney, pending its solution, must have been overwhelmed with the usual materials for its urinary function, which, instead of finding access to the corresponding ureter, remained in the entire capsule, and caused its increasing distention to the size it ultimately attained.

No remedy, beyond alleviation, could be reasonably attempted in such morbid circumstances; but, if the inflammatory excitement that originated the mischief had been duly repressed and overcome, the afflicting and destructive consequences that ensued might have been prevented. Inflammation, or immoderate excitement, is the hydra evil of health and life; and, if it be not seasonably resisted and subdued, it may lay the foundation of the most severe, insidious, and irreparable ailments incident to human nature.

A case, possessing a general resemblance to the preceding, lately occurred in the person of a gentleman of about thirty years of age, of a sanguineous temperament, and who, until this fatal illness, had been almost uniformly healthy. His disease at first assumed the character of typhous fever, which had a protracted duration, and entailed a destructive affection of one of the kidneys. During the prevalence of the fever, considerable pain had been experienced in the region of the kidneys, and also in that of the liver. It is probable, indeed, that the fever itself was originated by an inflammatory affection of one or both of these different organs. Typhous fever is never more intractable or durable than when connected with visceral inflammation, if that disease should not have been seasonably and adequately combated by vascular depletion. Blood-letting on these occasions is the most effectual remedy that can be employed: in fact, it would seem to be so indispensably necessary, that destructive mischief on the inflamed viscus cannot be prevented without its assistance.

In the case under consideration, convalescence was said to have occurred, but final recovery did not ensue. The patient was sent from London to the country, for the benefit of air, under circumstances very likely to terminate in a restoration of health. Soon after being in the country, a puriform fluid was voided, first in the natural course of the urethral canal, and afterwards through a small abscess that had formed and effected a passage from the membranous part of the urethra to the perineum. Nearly the natural quantity of water was secreted and discharged, and the chief complaint of pain was referred to the general cavity of the abdomen, which became extremely tense, and much distended. The enlargement was such, indeed, as to induce a suspicion of ascital accumulation of water; the test of fluctuation, however, was very obscure, yet sufficient, under the severe distresses of the patient from abdominal fullness, and in conformity to his importunate desire, to warrant resorting to the measure of tapping. This was performed cautiously, to obviate the risk that might have attended a less guarded introduction of the trocar. No water immediately followed the withdrawing of the trocar, but, by circumjacent pressure, and by inclining the body more particularly towards the canula, some fluid was made to flow, but not more than about a quart. The removal of this fluid afforded but little or no relief to the afflicting distention, which seemed to be hurtfully pressing against the stomach and diaphragm. The appetite had been rather keen than otherwise, but digestion proceeded heavily and imperfectly; the bowels also were torpid, and moved with difficulty. After suffering very acutely for about a week from the period of being tapped, the patient gradually sunk from progressive diminution of strength and final exhaustion.

The day after the decease, the cavity of the abdomen was inspected, the viscera of which did not present any striking morbid appearance. The stomach, intestines, liver, pancreas, and spleen, were for the most part free from disease, excepting an unnatural aspect of vascularity on the intestines and liver, denoting the inordinate irritation which had long existed in these parts. On extending the investigation to the kidneys, one of them appeared to be much enlarged, the other was of the natural size. An incision was made into that which seemed to be augmented, when it was found that its interior was excavated, and filled with a purulent fluid. Nearly the whole substance of the diseased kidney had undergone decomposition; and, if life had been sufficiently protracted, it is probable that its capsule, as in the case before recited, would have solely remained as the envelope, or containing part, of the puriform residuum of the dissolved kidney. The matter that had been thus enclosed was but slightly fetid, and amounted to perhaps about a pint in quantity. Its apparent quality was that of being well conditioned. Portions of it had, no doubt, entered into the ureter of the diseased kidney, and obtained an exit both at the urethral extremity of the penis, and by the new route that had been traced through the membranous part of the urethra and the perineum. The vent which took place in this direction from the suppurated kidney, prevented the degree of accumulation that would, probably, at length, have burst into the cavity of the abdomen.

The anomaly in this case is, that the region of the kidneys was not referred to as a chief source of complaint: indeed, not sufficiently to induce any suspicion of the renal organs being the seat of the prevailing disease. The urinary secretion, if not in natural quantity, was too considerable, and voided with a freedom that would not warrant an opinion that the kidneys were suffering under any organic affection. This almost healthy state of urinary secretion was performed by the sound kidney, evincing the importance of a duplicate provision for those vital functions that are more particularly liable to be disordered and impeded.

The suppurative, or rather ulcerative, process that demolished the substance of the kidney in this case, independently of the hurtful influence of purulent absorption, kept up an incessant state of morbid excitement throughout the system, under which vital power became so wasted and worn as to terminate in mortal exhaustion. It may be of practical importance to remark, that this patient had not been bled even in the early stage of the reputed typhous fever, that either commenced or terminated in the renal inflammation that proved destructive. Had the sanguiferous vessels been freely relieved of the congestive and distending plenitude which is apt, in typhoid disease, more particularly to oppress the visceral organs, an exemption might possibly have been afforded from the dreadful sequel that its omission probably occasioned. The insidiousness with which the parenchymatous structure of vital organs will assume inflammatory action, and its dangerous tendency in every instance in which it occurs, confers on the expediency of abstracting blood on these occasions a latitude of authority, that may be safely regarded as incontrovertibly just.

*Taunton; March* 18*th,* 1823.

